# Variations in schools’ commitment to health and implementation of health improvement activities: a cross-sectional study of secondary schools in Wales

**DOI:** 10.1186/s12889-016-2763-0

**Published:** 2016-02-10

**Authors:** Graham F. Moore, Hannah J. Littlecott, Adam Fletcher, Gillian Hewitt, Simon Murphy

**Affiliations:** DECIPHer, School of Social Sciences, Cardiff University, 1-3 Museum Place, Cardiff, CF10 3BD Wales UK

**Keywords:** Socioeconomic status, School, Adolescent, Child, Health behaviours, Inequalities

## Abstract

**Background:**

Interventions to improve young people’s health are most commonly delivered via schools. While young people attending the lowest socioeconomic status (SES) schools report poorer health profiles, no previous studies have examined whether there is an ‘inverse care law’ in school health improvement activity (i.e., whether schools in more affluent areas deliver more health improvement). Nor have other factors that may explain variations, such as leadership of health improvement activities, been examined at a population level. This paper examines variability in delivery of health improvement actions among secondary schools in Wales, and whether variability is linked to organisational commitment to health, socioeconomic status and school size.

**Methods:**

Of the 82 schools participating in the 2013/14 Health Behaviour in School-aged Children (HBSC) survey in Wales, 67 completed a questionnaire on school health improvement delivery structures and health improvement actions within their school. Correlational analyses explore associations of delivery of health improvement activity among schools in Wales with organisational commitment to health, socioeconomic context and school size.

**Results:**

There is substantial variability among schools in organisational commitment to health, with pupil emotional health identified as a priority by 52 % of schools, and physical health by 43 %. Approximately half (49 %) report written action plans for pupil health. Based on composite measures, the quantity of school health improvement activity was greater in less affluent schools and schools reporting greater commitment to health. There was a consistent though non-significant trend toward more health improvement activity in larger schools. In multivariate analysis deprivation (OR = 1.06; 95 % CI = 1.01 to 1.12) and organisational commitment to health were significant independent predictors of the quantity of health improvement (OR = 1.60; 95 % CI = 1.15 to 2.22).

**Conclusions:**

There is no evidence of an ‘inverse care law’ in school health, with some evidence of more comprehensive, multi-level health improvement activity in more deprived schools. This large-scale, quantitative analysis supports previous smaller scale, qualitative studies/process evaluations that suggest that senior management team commitment to delivering health improvement, and formulating and reviewing progress against written action plans, are important for facilitating the delivery of comprehensive interventions.

**Electronic supplementary material:**

The online version of this article (doi:10.1186/s12889-016-2763-0) contains supplementary material, which is available to authorized users.

## Background

Schools continue to be key settings for public health strategies aiming to improve health and reduce health inequalities for several reasons. First, the years young people spend at school are a formative period in their ‘health career’ [1], with socioeconomic inequalities in many health-risk behaviours, such as smoking, alcohol misuse and sedentary behaviour, emerging and becoming entrenched during this period [2–4]. Second, where education is provided universally, schools provide access to the vast majority of young people and thus have potential to improve health at a population level [5]. Third, the largest amount of public spending focussed on young people is typically made via national education systems, with staff professionally trained to support young people’s development. Fourth, there is now clear evidence that the school environment itself can influence young people’s health in various positive and negative ways [6]. Finally, there is growing evidence that promoting students’ health and wellbeing is synergistic with improving educational attainment [7].

For these reasons the World Health Organisation (WHO) developed the tripartite Health Promoting Schools (HPS) framework, focused not only on health education but also on promoting students’ health through the physical and social environment of school and involving families and wider communities in school-life [8, 9]. The HPS framework advocates the re-orientation of the whole school system towards health, with health improvement becoming a normalised part of what schools do, rather than a set of bolt-on activities. A systematic review of the effects of interventions adopting this HPS framework found that they produce small but significant public health benefits [8]. Another systematic review of the effects of modifying the school environment to improve students’ health also supported the use of the HPS framework [6]. Interventions which include such ‘higher-level’ environmental components also tend to be more cost-effective [10], and may be less likely to generate socioeconomic inequalities than individually focused, educational approaches [11].

However, to deliver significant public health gains requires HPS interventions to be adopted, implemented and maintained universally throughout national school systems [6]. This is far from straightforward. MRC guidance for evaluating complex interventions emphasises the need to consider whether complex interventions will be implementable on a wider scale throughout their development and evaluation [12]. However, evaluation practice has continued to be dominated by models in which complexity is conceived purely as a characteristic of the intervention, rather than of the systems into which interventions are to be delivered [13]. Interventions can perhaps best be understood as events within systems [14], or attempts to harness and re-orient existing system dynamics towards promoting health. Schools can be conceptualised as complex adaptive systems; their functioning is shaped by interactions among diverse and ever changing agents, while their ethos and network structures may support or impede the integration of new health improvement actions [15]. Hence, beginning to understand the complexities of delivering universal intervention approaches within school systems requires an understanding of what schools already do, and why.

To date, integration of HPS activities has been challenging in many contexts, possibly due to issues such as inadequate provision of supporting structures, resources and appropriate skills within the school setting [15, 16]. Qualitative studies exploring the implementation of interventions using the HPS framework have found that while incremental changes to a school’s practices, such as delivery of health education within the curriculum, can be enacted relatively quickly, more fundamental changes to the functioning of school systems, such as modifying a school’s environment or engaging parents and communities in health improvement activity, often prove more challenging [17–19]. However, variations in schools’ commitment to student health and their delivery of activities at a *population level* remain under-researched and under-theorised – for example, whether levels of commitment and delivery vary systematically according to schools’ socio-economic profiles or size, and what factors are associated with any variations in commitment to health and implementation across schools. Understanding population-level variations in schools’ commitment to student health and implementation of health improvement activities is vital to ensure that health inequalities are not exacerbated – rather than reduced – via investment in school health.

There is some evidence from Wales that pupils attending more affluent schools tend to report healthier behaviours than those attending poorer schools after adjustment for family-level socio-economic status (SES), while gradients by family-level SES are greatest in affluent schools [20]. Neo-materialist theories of health inequalities argue that the provision of public and social services, such as education, and their capacity to improve health, varies systematically according to differences in communities’ socio-economic characteristics and this, in turn, partly explains the extent of the social inequalities observed in health outcomes in countries such as the UK [21]. These theories address some of the limitations of traditional, cruder material explanations, situating health inequalities in the context of public policies and recognising the social importance of place for shaping institutions, local cultures and individuals’ behaviours [22].

Neo-materialist theories also draw attention to the ‘inverse care law’, whereby the availability of good medical or social care has been found to vary inversely with the need of the population served [23]. An inverse care law in relation to provision of school health improvement activity could potentially offer one explanation for the aforementioned socioeconomic inequalities in health behaviours previously observed between schools in Wales [20]. However, whether commitment to health or delivery of school health improvement activity are patterned by school-level socioeconomic compositions has yet to be tested. Empirical evidence on the role of school size on education and health outcome is also a major blindspot at present; what little evidence there is of effects on educational outcomes remains equivocal [24]. Larger schools are perhaps likely to possess more complex system-level structures, including larger numbers of sub- and supra- systems [15], and as such achieving change may be more difficult. Conversely, the greater diversity in agents within larger systems may mean that there is a broader skills mix to draw upon in implementing new actions, or a greater number of staff committed to student health.

Previous empirical studies have identified some factors that may explain why adoption and implementation of health improvement activities varies across schools. A review of the implementation literature suggested the following eight factors facilitated the implementation of health improvement activities in schools: preparation and planning; policy support; opportunities for professional development; support and buy-in from leadership; supportive relational and organisational context; student participation; partnership working; and the potential for sustainability [25, 26]. Relating to Rowling and Samdal’s theme of ‘support and buy in from leadership’, the role of ‘champions’ in engaging and motivating other staff, parents and students, and involving the whole school community in health improvement, has been emphasised by advocates of the HPS approach [27, 28]. Drawing on qualitative data collected in Scottish schools, Inchley et al. [17] argue that where this role is assigned to a senior figure, a school is more likely to achieve health promoting school status, due to the greater degree of leverage and influence such individuals hold over the system as a whole. Other studies have also identified the importance of senior management commitment to student health through the development of action plans, and regularly reviewing schools’ progress against them via local data [29, 30]. However, no studies have empirically tested the importance of these factors across a large number of schools.

To address these empirical gaps, this paper draws on school-level data from a large, representative sample of secondary schools in Wales. It begins by mapping variability in the priority given to student health and their management systems and structures for health improvement, examining whether student health is identified as a priority area, the presence of regularly reviewed written action plans for health, the seniority of individuals tasked with leading health improvement activity and the use of data to inform health improvement. Our analyses test the ‘inverse care law’ through use of school-level data on social deprivation, and also whether organisational commitment and management vary systematically according to school size. Subsequently, the paper focuses on the extent and nature of variability in the delivery of activities according to specific domains of health improvement activity, including food and fitness, substance use, and mental and emotional health. Variations by organisational commitment to health, school-level deprivation and school size are examined for each of these areas of activity.

## Methods

### Participants and sampling

The Health Behaviour in School-aged Children survey (HBSC) in Wales is a cross-sectional study of school students aged 11–16 [31]. Wales is one of a number of countries participating in the HBSC study internationally. In the 2013/4 HSBC Wales survey, a nationally representative sample of 82 secondary schools was recruited via stratified, random sampling. All maintained and independent secondary schools in Wales were stratified by local authority and eligibility for free school meals. Schools were selected using probability proportionate to size *and* there was an element of disproportionate stratification to allow analysis at Local Health Board level.

For the 2013/14 survey in Wales, schools were also asked to complete questionnaires on the school environment and school health improvement actions. This questionnaire was completed by a staff member of 67 of the 82 HBSC survey schools. The questionnaire was sent to head teachers, who were asked to nominate a member of staff to complete it. In 8 cases, the questionnaire was completed by a head teacher (or acting head teacher), in 18 by a deputy head teacher and in 21 by an assistant head teacher. Six each were completed by heads of year or by “healthy school coordinators”, and 8 by another unnamed member of staff. Ethical approval was obtained from the Cardiff University School of Social Sciences Research Ethics Committee. Head teachers were provided with full information on the HBSC survey, and provided written consent for their school to participate, before completing the School Environment Questionnaire or nominating another member of staff to do so on their behalf.

### Measures

#### School size and free school meal entitlement

Details on the number of students on the school roll and the percentage of children entitled to free school meals were provided by Welsh Government to measure school size and school-level deprivation.

#### The school environment questionnaire

The questionnaire completed by school staff included questions regarding organisational structures for delivery of health improvement, and the presence, breadth and depth of school health improvement activities. The questionnaire included items from school surveys in Canada [32] and was tailored to include priority topics in the Welsh context. Schools were asked to answer in relation to years 7–11 (compulsory education years only). The following variables are used in analysis.

#### Organisational commitment to student health

Schools were asked to select up to 4 areas which had been prioritised by the senior management team in the past 2 academic years from a list of 9 areas, including student emotional and mental health, student physical health, and staff health as well as items on educational performance and school environment. A score of 0 was assigned if neither student health item was selected, 1 if one was, and 2 if both were. Schools were also asked if they had a written action plan for student health, and how often this was reviewed. A score of 0 was assigned if there was no action plan, 1 for action plans that were reviewed less than once a year and 2 if there was a written policy which was reviewed annually. These items were summed to form an ordinal scale scored from 0 (lowest level of organisational commitment to health) to 4 (highest level of organisational commitment to health).

#### Leadership of health improvement

Schools were asked if they had a single strategic lead for health initiatives, with options of yes, different leads for different areas of health, or no one leads. Those who stated that they had a single strategic lead were asked what this person’s job role was (e.g., deputy headteacher, class teacher). A combined variable was created which divided schools into those with a single senior lead (i.e., assistant headteacher or above), a single more junior lead, multiple leads or no lead.

#### Use of data to inform school health

Schools were asked whether they used information or data to inform or make changes to school health policy, with response options of ‘yes, we use information and data as much as possible’, ‘yes, we sometimes use information and data’ or ‘no’. Schools were also presented with a list of possible data sources, including internal student and staff surveys or routine school data and asked which of those data sources they used.

#### Nutrition

Schools were asked whether they had a healthy eating, or food and fitness policy, with options of yes, currently in development or no. Schools were also asked whether this policy covered a range of extracurricular activities or food brought into the school by students. These items were manipulated into a 5 point ordinal variable (i) no policy, (ii) policy in development, (iii) policy covering food provision, (iv) policy covering food provision plus either extracurricular activities or food brought in by students, (v) policy covering food provision, extracurricular activities and food brought in by students). Schools were also asked whether healthy items were promoted in their canteens, with options of ‘yes – always’, ‘yes – sometimes’ or ‘no’. Further items asked schools (i) whether they ran extracurricular nutrition education sessions (e.g., cooking classes) with options of ‘regularly’, ‘ad-hoc events’ or ‘no’ and (ii) whether parents were invited to take part in these sessions.

#### Physical activity

Schools were asked to indicate the amount of time per week students in each year received Physical Education (PE) within the curriculum, with items summed to give an ordinal score. The availability of extra-curricular sport was assessed through asking how many days per week students could participate in extra-curricular sports in autumn and in summer, with items summed to give an ordinal score. The availability of sport facilities was assessed by presenting schools with a list of facilities (e.g., gymnasium, dance/fitness studio, swimming pool, running track) and asking whether these were available for students to use (i) during PE, (ii) during lunch breaks or (iii) after school. The total number of facilities available at each time was calculated by summing responses for each item. Due to strong correlations between availability of facilities in PE, lunchtime and after school (*r* = 0.66 to 0.88), these 3 items were summed to form a combined scale (alpha = 0.81). To capture activity relating to active transport, the questionnaire included a list of strategies (e.g., identifying safe walking and cycle routes, walking promotions, cycle proficiency training) to promote active travel which, if any, they used. A sum score was created for the number of strategies (0–7) used to promote active travel to school. Schools were also asked whether they had partnerships with families to promote physical activity, and whether parents were involved in delivery of extra-curricular sport; these two items were summed to provide an indicator of parental and family involvement in physical activity promotion.

#### Substance use

Schools were asked whether they had a written policy on smoking and tobacco use (yes, currently in development, or no), and how frequently this was reviewed (at least once a year, less than once a year, never been reviewed). These items were combined into an ordinal item with a score of 0 = no policy, 1 = policy in development, 2 = policy reviewed less than annually, 3 = policy reviewed at least annually. Schools were also asked whether smoking was prohibited (i) on school grounds during school hours, (ii) on school outside of school hours, (iii) in private vehicles on school grounds and (iv) during school events off school grounds. Each item was given a score 0 or 1, and summed to provide a score of 0–4 (indicating the coverage of school smoke-free policy). Schools were also asked whether all year groups received tobacco education, alcohol education or drug education, and if not, which year groups were excluded. The number of year groups receiving education for each topic was calculated, and summed to form a composite scale for coverage of substance misuse education (alpha = 0.82). Data were missing for 4 schools on this variable, and hence prior to creation of composite scores, schools were divided into those who provided tobacco, alcohol and drug education to all year groups vs those who did not (including those with missing values).

#### Mental health and wellbeing

Schools were asked whether they had a written policy on mental health and wellbeing (yes, currently in development, or no), and how frequently this was reviewed (at least once a year, less than once a year, never been reviewed). These items were combined into an ordinal item with a score of 0 = no policy, 1 = policy in development, 2 = policy reviewed less than annually, 3 = policy reviewed at least annually. Schools were also asked whether they had a policy on bullying, and whether cyber-bullying was included within this.

#### Sexual health and relationships

Schools were asked which year groups received sex and relationships education, with a score of 1–5 generated by summing responses for each year group. For this variable, data were missing for 10 schools. As sexual health and relationships education (SHRE) is mandatory, for correlational analysis we did not assume that missing data represented non-delivery. However prior to creating composite measures (i.e., total education score), this variable was divided into schools which stated that they delivered SHRE to all year groups vs those who did not (including “missing”).

#### Personal and social education (PSE)

Time devoted to PSE was assessed by asking how many minutes on average PSE was delivered to each year group each week, with responses summed to provide an ordinal item for total PSE time. For this variable, data were missing for 7 schools. As PSE is mandatory, for correlational analysis, we did not assume that missing data represented non-delivery. However prior to creating composite measures (i.e., total education score), this variable was divided into schools which stated that they delivered SHRE to all year groups vs those who did not (including “missing”).

#### Composite measures of actions by HPS level

The above actions were divided into three categories of (1) education/curriculum delivery, (2) change to social and physical environments/ethos (e.g., policy implementation), and (3) parental/family involvement and summed to form 3 composite measures to indicate the extent of variability health improvement activity in these 3 domains. Schools were then divided at the median for each of these 3 variables, and assigned a score of 0 (low) or 1 (high). These 3 binary scores were summed to form a scale scored 0 to 3, indicating the number of levels of action for which the school scored above the median.

### Statistical analysis

For key variables in relation to school health improvement structures and activities, frequencies and percentages are presented. In order to test associations between ordinal variables, Spearman’s Rank correlation coefficients are used, while Mann Whitney U-tests are used to examine associations between nominal and ordinal variables (see Additional file 1). Finally, for the composite measure of consistency with the HPS framework, ordinal logistic regression analysis is conducted to examine the extent to which this is independently associated with deprivation, school size and organisational commitment to health. For many variables, the questionnaire asked participants to tick a box if it applied, and hence the absence of a response was interpreted as non-delivery. Assumptions relating to missing data for a small number of key variables are described above. As a sensitivity analysis, analyses for composite variables were run for complete data only and with missing values imputed. As results were consistent, only the latter are reported.

## Results

### Organisational commitment to student health

Table 1 below lists the percentage of schools indicating having prioritised each area for improvement in the past 2 years. Educational attainment was selected by the largest proportion of schools, followed by Estyn report and banding^1^, selected by 2 in 3. Half of schools identified student mental/emotional health as a priority area, while slightly less than half identified student physical health. The only choices less likely than student physical health to be selected were relationships with local community, staff health and provision of extra-curricular activities. However, most schools (*n* = 41; 61.2 %) selected at least one of the two options relating to pupil health, while one in 3 (*n* = 23; 34.3 %) selected both. Half of schools (*n* = 33; 49.3 %) reported that they had a written school health action plan, or school health targets in place. In 24 of these schools (72.7 %), progress against action plans was reviewed at least annually. Schools divided almost equally across the 5 levels of the ‘organisational commitment to health’ measure derived from the above 2 measures, indicating substantial variability in organisational commitment to student health throughout Wales.

**Table 1:** Number and percentage of schools indicating each topic area as one of their 4 top priorities (*n* = 67)

	Frequency (percentage)
Educational attainment	63 (94.0)
Estyn report and banding	46 (68.7)
Physical condition of school buildings	35 (52.2)
**Student mental/emotional health**	**35 (52.2)**
Relationship with parents	31 (46.3)
**Student physical health**	**29 (43.3)**
Relationship with local community	22 (32.9)
Staff health	20 (29.9)
Provision of extra-curricular activities	18 (26.9)

Items relating to health are highlighted in bold

### Leadership of health improvement

Most schools (*n* = 40; 59.7 %) reported having a single lead for school or student health initiatives. In most cases (*N* = 23), this was a senior member of staff (i.e., a deputy or assistant head teacher). Among schools who reported having multiple leads, a minority (9; 34.6 %) reported having written action plans. Small majorities of schools had a written action plan if there was a single junior (11; 64.7 %) or senior lead (13; 56.5 %) responsible for school health.

### Use of information and data to inform health improvement

When asked if their school makes use of information and data to update its policies on school health, a vast majority said that they either use data as much as possible (47; 70.2 %) or sometimes (14; 20.9). However, the percentage who identified at least one specific source of data used was lower (43; 64.2 %). The most commonly used data were routinely collected school data, while 1 in 7 reported that they used either internal student surveys or other surveys. When asked who these data were used by, in most cases (32; 74.4 %), schools indicated that they were used by the Healthy Schools Coordinator.

### Institutional characteristics and organisational commitment to health

There was little correlation between school-level affluence (FSM entitlement) and commitment to health (*r* = −0.00), indicating that organisational commitment to health did not vary according to the socioeconomic composition of the school. There was a larger positive correlation between school size and commitment to health (*r* = 0.17) indicating a trend towards stronger commitment in larger schools, although again this was not significant. There was a small and non-significant correlation of FSM entitlement with school size.

### School health actions

The nature and extent of school health improvement policies and actions delivered in Wales, and how they vary across school nationally, are indicated in Table 2. Some appear almost universally present across schools. For example, almost all schools reported having a written anti-bullying policy (no schools stated that they did not, although 4 did not answer this question), while a vast majority reported a written tobacco policy, and almost all said that healthy food options are promoted in their canteen. However, there were numerous areas of substantial variability. A third of schools reported that they did not have a written policy on mental health and wellbeing. While almost two-thirds of schools reported having a healthy eating policy, schools were divided as to whether this covered food brought in or food consumed in extracurricular activities. Sex and relationships education is provided to all year groups in two-fifths of schools, while in a third of schools it is provided only to 1 or 2 year groups. PE time within the curriculum drops substantially as children enter GCSE years (Year 10) in many schools, with 77 % of young people receiving more than 90 min of PE in Year 9, falling to 27 % in Year 10. Most schools deliver more than 30 but less than 90 min of PSE per week, though as children enter Year 10, some schools reduce PSE time, while others increase it. A minority of schools report involving parents in the delivery of extracurricular physical activity or having partnerships with families to support physical activity. Most schools who report delivering extra-curricular food education programmes report that parents are rarely or never involved in these.

**Table 2:** Implementation of school health improvement activities by schools throughout Wales (*n* = 67)

			Number	Percentage
Nutrition	Healthy eating policy	No policy	9	13.4
		Policy in development	10	14.9
		Written policy	12	17.9
		Covering extracurricular activity or food brought in	14	20.9
		Covering extracurricular activity and food brought in	22	32.8
	Healthy food promoted in canteen	No	3	4.5
		Sometimes	15	22.4
		Always	49	73.1
	Extra-curricular nutrition education programmes	None	13	19.4
		Adhoc events	27	40.3
		Regularly	27	40.3
	Parents invited to take part in these programmes	Never	25	46.3
		Rarely	10	18.5
		Sometimes	16	29.6
		Always	3	5.6
Physical activity	PE time within curriculum (Year 9)	<60 min	5	7.6
		60–89 min	10	15.2
		90–119 min	25	37.9
		>120 min	26	39.4
	PE time within curriculum (Year 10)	<60 min	12	18.2
		60–89 min	36	54.5
		90–119 min	9	13.6
		>120 min	9	13.6
	Number of days extracurricular sports offered	1 to 2	4	6.1
		3 to 4	22	33.3
		5	40	60.6
	Number of strategies to promote active travel	Median and IQR	3	(2 to 4)
	Number of sport facilities available for PE	Median and IQR	7	(5 to 8)
	Number of sport facilities available at lunchtime	Median and IQR	6	(4 to 7)
	Number of sport facilities available after school	Median and IQR	6	(5 to 7)
	Involvement of parents in delivery of extracurricular PA	Yes	9	13.4
	Partnerships with families to promote PA	Yes	11	16.4
Substance use	Presence of written smoking policy	No	9	13.4
		In development	2	3.0
		Yes - reviewed less than annually	31	46.3
		Yes - reviewed annually	25	37.3
	Coverage of smoke-free policy	On school grounds during school hours	54	80.6
		On school grounds outside of school hours	43	64.2
		Private vehicles on school grounds	30	44.8
		School events off school grounds	45	67.2
	Substance education to all years	Tobacco	44	65.7
		Alcohol	45	67.2
		Drugs	51	76.1
Emotional health	Written policy on mental health and wellbeing	No	24	35.8
		In development	15	22.4
		Yes - reviewed less than annually	11	16.4
		Yes - reviewed annually	17	25.4
		Not stated	4	6.0
	Policies on bullying			
		Yes (in mental health policy)	20	29.9
		Yes (in another policy)	43	64.2
	Cyber bullying included within bullying policy?	Yes	59	93.7
		1	6	10.5
	Number of year groups received sex and relationships education	2	12	21.1
		3	8	14.0
		4	7	12.3
		5	24	42.1
Personal and social education	Weekly time committed to PSE (Year 8 and 9)	None	3	4.8
		less than 30 min	3	4.8
		30–59 min	39	62.9
		60–89 min	16	25.8
		90 min or more	1	1.6
	Weekly time committed to PSE (Year 10)	None	2	3.2
		less than 30 min	5	8.1
		30–59 min	31	50.0
		60–89 min	19	30.7
		90 min or more	5	8.1

### Correlates of school health improvement

As indicated in Table 3, school size had small positive correlations with most health improvement activities indicating a trend toward more health improvement activity in larger schools, though in no cases was this significant, approaching significance only for coverage of healthy food policy and promotion of active travel. Similarly, for schools with higher levels of FSM entitlement, there were small positive correlations for most measures, indicating a non-significant trend toward more health improvement activity in more deprived schools. Most school health related actions increased substantially with increased organisational commitment to health, including the presence and coverage of policies around healthy eating, time dedicated to extracurricular sport, availability of facilities for extracurricular sport, promotion of active travel, coverage of smoke-free policies and the number of year groups receiving education on substance misuse. Reported use of data was not significantly associated with any health related activity except for a greater likelihood of reporting a policy for emotional or mental health (see Additional file 1), nor was leadership of school health (i.e., whether assigned to a single junior/senior individual or to multiple leads). For composite measures, organisational commitment to health was associated with increased environment/policy intervention, with a near significant increase in family involvement. Free school meal entitlement was associated with delivery of health education, with a non-significant trend towards increased environmental/policy intervention. For the composite measure of consistency with the HPS framework, there were significant univariable associations of free school meal entitlement and organisational commitment, and a non-significant trend toward more activity in larger schools. In a multivariate ordinal regression model, deprivation (OR = 1.06; 95 % CI = 1.01 to 1.12) and organisational commitment to health remained significant (OR = 1.60; 95 % CI = 1.15 to 2.22).

**Table 3:** Correlations between deprivation, school size, organisational commitment to health and health improvement activities in secondary schools in Wales (*N* = 67)

		FSM entitlement	School size	Organisational commitment to health
Healthy eating	Healthy eating policy	0.06	0.25*	0.41**
	Healthy food promoted in canteen	−0.04	0.03	0.35**
	Extra-curricular nutrition education programmes	0.16	0.01	−0.04
	Parental involvement in nutrition education	0.01	0.03	0.14
Physical activity	Number of days extracurricular sports available	0.06	0.17	0.27**
	Number of sport facilities	0.03	0.19	0.33**
	PE time within curriculum	0.15	0.12	−0.13
	Number of strategies to promote active travel	−0.08	0.24*	0.22*
	Involvement of parents and families	−0.01	0.20	0.19
Substance use	Presence of written policy on smoking	0.21*	0.10	0.07
	Coverage of smoke-free policy	−0.08	0.05	0.25**
	Number of year groups receiving substance misuse education	0.23*	0.03	0.25**
Mental/Emotional health	Written policy for emotional health	0.23*	−0.06	0.16
Sex and relationships	Number of year groups receiving sex and relationships education	0.15	−0.09	0.25*
Personal and Social Education	Weekly time dedicated to PSE	0.30**	0.08	0.08
Composite measures	Sum of education items	0.30**	0.05	0.19
	Sum of environment/policy items	0.16	0.23*	0.46**
	Sum of family involvement items	0.04	0.12	0.18
	Health Promoting Schools composite score	0.30**	0.20	0.40**

*p<0.10

**p<0.05

## Discussion

This study demonstrates a marked diversity in organisational commitment to pupil health improvement among secondary schools in Wales; about half identify student health as a priority area, with more prioritising student mental and emotional health (52.2 %) than physical health (43.3 %). About half report that they have written action plans in place for student health. Consistent with earlier studies of the HPS model which cite leadership, school structures and policies, information, collaboration, resources and political support as factors that aid implementation [33], organisational commitment to student health was consistently correlated with a wide range of health improvement activity. Contrasting with earlier literature [17], whether leadership responsibility was assigned to a more senior or junior figure was not correlated with school health improvement actions. Hence, it may be that whether staff assigned responsibility for school health are fully backed by senior management is more critical than whether they are themselves senior figures within the school.

While it is perhaps encouraging that half of schools view pupil health as a priority, it remains concerning both that student health appears to be given limited priority by senior management within a large proportion of schools, and that this appears to translate into more limited delivery of health improvement activity. Viewing school-based interventions as events within complex systems [13, 14], it is perhaps likely that in schools which see health improvement either as a low priority activity or as something which conflicts with educational goals, existing system structures will block the flow of new information and activities throughout the system [15]. Hence, the universal delivery of such approaches needed to realise their potential public health impact may prove difficult to achieve, unless concerted efforts are made to persuade many schools of the value of promoting pupil health. A tendency for health and education to be viewed as conflicting priorities is perhaps evidenced by findings such as the marked drop off in weekly time devoted to physical education once pupils entered the final 2 years of compulsory education (the time period during which school-leaving qualifications are pursued in UK schools). However, this approach to school management and leadership is paradoxical in light of the educational benefits of promoting student health [7].

Notably however, while practices varied substantially between schools, in contrast to neo-materialist explanations of health inequality – which emphasise the role of the unequal distribution of services and resources in perpetuating inequality there was no evidence of an ‘inverse care law’ [23] – health improvement activity was neither prioritised nor delivered to a lesser extent in poorer schools. Indeed, there was a tendency for a greater volume of health improvement activity in schools with more deprived intakes. Hence, there is some suggestion that schools in poorer areas may have more comprehensive health improvement actions in place, perhaps as a means of countering the effects of socioeconomic deprivation on pupil health. Hence, while between school SES inequalities in health-related behaviours have been reported in Welsh schools [20], these are unlikely to be explained by systematic variability in school commitment to pupil health or in the quantity of health improvement activity.

It is perhaps plausible that other features of more deprived school contexts impede the ability of health improvement activity to work as effectively, or that a higher volume of activity is delivered, though not with greater quality. Our recent systematic review suggests that universal interventions delivered via schools are equally likely to narrow or widen inequality [34]. An established body of educational literature shows that schools in poorer areas face greater challenges in recruiting and retaining high quality teaching staff, with implications for teaching quality [35]; implications of issues such as these for the delivery of health improvement have not been explored. Notably, though there is some evidence that more structural interventions may be necessary to reduce inequality [34], deprivation was more strongly associated with increased health education than with environmental intervention or family involvement. There was also no evidence of an association between school size and the volume of health improvement actions, although a consistent trend in the direction of more activity in larger schools.

The study benefits from sampling of a large and nationally representative range of schools throughout Wales. However, limitations include the reliance on self-report measures of school health improvement actions, as well as a focus on the quantity of health improvement activity rather than its quality or the extent to which the manner in which it was delivered is grounded in evidence. It may be for example that having a small number of well implemented evidence-informed programmes to improve pupils’ health is more beneficial than having a large number of different activities. The questionnaire was also not designed explicitly to capture consistency with an HPS framework, and hence measures such as parental and community involvement are limited and may have overlooked a wide range of additional activities delivered within Welsh Schools. Though widely used [34], there has been some criticism of the use of free school meal entitlement as a marker of SES.

## Conclusion

Nevertheless, the study highlights some important challenges in attempting to implement whole-school health improvement approaches within secondary schools in Wales. Investment in HPS interventions has potential for population-level improvement, with no evidence of unequal distribution of resources by deprivation level. Variations in health improvement activities appear to be linked to a substantial degree of variability in senior management commitment within schools. Hence, government policies, public health strategies and health improvement interventions need to ensure senior management level buy-in, perhaps via mechanisms such as including health related targets within inspection frameworks, or further developing evidence bases regarding the synergy between health and educational outcomes to assist in persuading school stakeholders of the importance of their role in promoting pupil health. While the present study focused on the quantity of health improvement activity, it made no assessment of its quality. Subsequent analyses will therefore include multi-level analyses to understand associations between the quantity of existing health improvement actions at the school level and health-related outcomes at the pupil level. It may be for example that even where there is a strong will to improve pupil health, the actions selected to achieve this are not effective. In addition, future research could usefully explore how socioeconomic contexts impact on the quality of implementation of health improvement activity, or young peoples’ interaction with health improvement activities. Developing and testing effective mechanisms of working with schools to identify appropriately tailored evidence-informed health improvement actions to address health needs within their schools is a priority for future research.

## Additional file

Additional file 1:
**Data use and health improvement action – sum ranks and kruskal wallis tests.** (DOCX 15 kb)

## Abbreviations

SES: Socioeconomic status
HPS: Health promoting schools
HBSC: Health behaviour in school-aged children
MRC: Medical Research Council

## Footnotes

1 All maintained schools in Wales are regularly assessed by Estyn, the education and training inspectorate. A publicly available report is published after each inspection. Banding was a Government system of identifying schools in need of support based on their performance and pupil attendance. All maintained secondary schools in Wales were placed in one of five bands.
