# Supplementary material for: Variations in schools’ commitment to health and implementation of health improvement activities: a cross-sectional study of secondary schools in Wales
Source: BMC Public Health. 2016 Feb 10;16:138. doi: 10.1186/s12889-016-2763-0 (PMC4750183; doi:10.1186/s12889-016-2763-0)
Supplement: Additional file 1: — Data use and health improvement action – sum ranks and kruskal wallis tests. (DOCX 15 kb) [file 12889_2016_2763_MOESM1_ESM.docx]

**Data use and health improvement action – sum ranks and kruskal wallis tests**

|  |  | **No (n=24)** | **Yes (n=43)** | **Chi2(p)** |
| --- | --- | --- | --- | --- |
| Healthy eating | Healthy eating policy | 786.5 | 1491.5 | 0.96 (0.33) |
|  | Healthy food promoted in canteen | 813 | 1465.0 | 0.00(0.97) |
|  | Extra-curricular nutrition education programmes | 892 | 1386 | 0.98(0.32) |
|  | Parental involvement in nutrition education | 786 | 1492 | 0.15(0.70) |
| Physical activity | Number of days extracurricular sports offered | 707.5 | 1437.5 | 0.07 (0.80) |
|  | Number of sport facilities | 783 | 1495 | 0.19 (0.67) |
|  | PE time within curriculum | 735 | 1476 | 0.85 (0.36) |
|  | Number of strategies to promote active travel | 862.5 | 1415.5 | 0.37 (0.54) |
|  | Involvement of parents and families | 738.5 | 1539.5 | 1.03(0.31) |
| Substance use | Presence of written policy on smoking | 734 | 1544 | 1.15 (0.28) |
|  | Coverage of smoke-free policy | 831 | 1447 | 0.04(0.84) |
|  | Number of year groups receiving substance misuse education | 633 | 1383 | 2.16(0.14) |
| Mental / emotional health | Written policy for emotional health | **654** | **1624** | **4.49 (0.03)** |
| Sex and relationships | Number of year groups receiving sex and relationships education | 494 | 1102 | 3.13 (0.08) |
| Personal and Social Education | Weekly time dedicated to PSE | 553 | 1277 | 0.80 (0.37) |
| Composite measures | Sum of education items | 669 | 1609 | 3.70 (0.05) |
|  | Sum of environment/policy items | 721 | 1490 | 0.44 (0.50) |
|  | Sum of family involvement items | 738 | 1540 | 1.04 (0.31) |
|  | Health Promoting Schools composite score | 690 | 1521 | 1.17 (0.28) |

|  |  | **Multiple** | **Junior** | **Senior** | **Chi2(p)** |
| --- | --- | --- | --- | --- | --- |
| Healthy eating | Healthy eating policy | 720 | 645.5 | 845.5 | 3.97(0.14) |
|  | Healthy food promoted in canteen | 781 | 547 | 883 | 2.42(0.29) |
|  | Extra-curricular nutrition education programmes | 860.5 | 611 | 739.5 | 0.40 (0.82) |
|  | Parental involvement in nutrition education | 908 | 558.5 | 744.5 | 0.24 (0.89) |
| Physical activity | Number of days extracurricular sports offered | 682.5 | 566 | 831.5 | 5.00(0.08) |
|  | Number of sport facilities | 790.5 | 712 | 708.5 | 4.37(0.11) |
|  | PE time within curriculum | 789.5 | 484.5 | 871 | 2.63 (0.27) |
|  | Number of strategies to promote active travel | 814 | 596.5 | 800.5 | 0.56 (0.76) |
|  | Involvement of parents and families | 931 | 463.5 | 816.5 | 2.42 (0.29) |
| Substance use | Presence of written policy on smoking | 876.5 | 595 | 739.5 | 0.22 (0.90) |
|  | Coverage of smoke-free policy | 776.5 | 601.5 | 833 | 1.56(0.46) |
|  | Number of year groups receiving substance misuse education | 726 | 493 | 734 | 0.04(0.98) |
| Mental / emotional health | Written policy for emotional health | 928 | 559 | 724 | 0.61 (0.74) |
| Sex and relationships | Number of year groups receiving sex and relationships education | 541 | 399.5 | 599.5 | 0.89 (0.64) |
| Personal and Social Education | Weekly time dedicated to PSE | 652.5 | 417.5 | 700 | 0.49(0.78) |
| Composite measures | Sum of education items | 817.5 | 489.5 | 904 | 3.423 (0.51) |
|  | Sum of environment/policy items | 747 | 617.5 | 780.5 | 2.77 (0.25) |
|  | Sum of family involvement items | 940 | 490.5 | 780.5 | 1.51(0.47) |
|  | Health Promoting Schools composite score | 944 | 500 | 767 | 1.33(0.52) |
